# 气相色谱-串联质谱法同时测定化妆品中28种香料成分

**DOI:** 10.3724/SP.J.1123.2022.03043

**Published:** 2023-01-08

**Authors:** Dunming XU, Yifeng WU, Yifan WANG, Fangfang CHEN, Shudi ZHANG, Guoyin LAI

**Affiliations:** 1.厦门海关技术中心, 福建 厦门 361026; 1. Technical Center of Xiamen Customs, Xiamen 361026, China; 2.厦门医学院, 福建 厦门 361023; 2. Xiamen Medical College, Xiamen 361023, China; 3.广东铭康香精香料有限公司, 广东 广州 510530; 3. Guangdong Wincom Flavors & Fragrances Co., Ltd., Guangzhou 510530, China

**Keywords:** 气相色谱-串联质谱法, 香料, 化妆品, gas chromatography-tandem mass spectrometry (GC-MS/MS), fragrances, cosmetics

## Abstract

随着化妆品种类的日益增多,化妆品中的香精香料成分受到了越来越多人的关注,建立化妆品中香精香料成分的分析测定方法是消除人们担忧最有效的措施之一。该研究建立了气相色谱-串联质谱(GC-MS/MS)同时测定化妆品中28种香料残留的方法。样品由甲醇提取,含油脂较多的经中性氧化铝固相萃取小柱净化,色素多、基质复杂的由QuEChERS净化,待测物采用GC-MS/MS测定,以保留时间和特征离子对定性,外标法定量。实验结果表明,分析物质的检出限(*S/N*=3)为2~20 μg/kg,定量限(*S/N>*10)为5~50 μg/kg。28种物质分别在1~100、2~200、4~200、10~1000 μg/L范围内线性关系良好,分析物质的线性相关系数(*r*^2^)>0.999。在空白样品中添加28种香料,添加水平为50~500 μg/kg时,回收率范围为71.3%~120.4%, RSD范围为1.5%~14.6%。该方法可对28种香料成分进行准确测定,不但可以有效排除复杂基体的干扰,而且简单、灵敏、稳定,能够很好地应用于化妆品中香料的含量测定。通过该方法对16种化妆品中的香精香料成分进行测定,发现12种化妆品中均检出相关香料成分,说明化妆品中的香精香料安全应引起重视。

## Abstract (en)

With the increasing number of cosmetic products, their flavor and fragrance components are receiving greater and greater attention. Establishing an analytical method of determining these components in cosmetics is one of the most effective measures to eliminate consumers’ concerns. In this study, a method for the simultaneous determination of 28 fragrance residues in cosmetics by gas chromatography-tandem mass spectrometry (GC-MS/MS) was developed. The samples were extracted using methanol and those containing more oil and grease were purified using a neutral alumina solid-phase extraction column, whereas those with more complex compositions were purified by QuEChERS. The analytes in the samples were measured by GC-MS/MS, characterized using their retention times and characteristic ion pairs, and quantified with an external standard. The respective limits of detection (LODs, *S/N*=3) and quantification (LOQs, *S/N*>10) of the compounds were in the ranges 2-20 and 5-50 μg/kg. The linearities of the concentration curves of the 28 substances were good in the ranges 1-100, 2-200, 4-200, and 10-1000 μg/L, and the correlation coefficients of the quantitative ion pairs were >0.999. Twenty-eight fragrances were added to blank samples at spiked levels of 50-500 μg/kg, and the recoveries ranged from 71.3% to 120.4%, with RSDs of 1.5%-14.6%. The method could be applied in the determination of fragrances in cosmetics because it was simple, sensitive, and stable and could effectively exclude the interferences of complex matrices. The method was used to determine the fragrance components in 16 cosmetic products, and some fragrance components were detected in 12 samples. Increased attention should be paid to the safeties of fragrances and flavors used in cosmetics.

近年来,人们的物质需求在日益增长,这点在化妆品市场中得到了很好的体现,化妆品行业也成为国民经济发展最快的行业之一^[1]^。从市场规模来看,2020年中国化妆品市场规模达到5000亿元,预计到2025年将达到10000亿元;从市场渠道来看,2020年中国化妆品市场零售额增速为9.5%,成为非必需消费品中增速较快的品类^[2]^。巨大的市场发展潜力对化妆品产品提出了更高的要求^[3]^。由于化妆品在使用过程中与人体肌肤直接接触^[1,4]^,因此化妆品的安全性受到人们的广泛关注,而其中一部分化妆品的安全性问题实质上是化妆品中添加的香料成分的安全问题。李杨杰等^[5]^就对化妆品中73种常见的禁用物质进行了测定,结果表明在多种化妆品中均检出了禁用物质,这也正说明化妆品的安全问题不容小觑。香料,包括天然香料和合成香料,是指从带香物质中提取或以人工合成方法得到的致香物质的总称^[6]^。因具有气味芳香、自然清新的特点,香料被广泛添加到各种日常生活用品中^[7]^,如洗衣液、清洁剂、调味品等,更是化妆品中的重要组成部分。随着香料在化妆品中的广泛应用,其安全性问题也逐渐显露,其中香料过敏问题尤为多见,占到全部化妆品过敏案例的1/3以上^[7]^。致敏性香料主要包括芳香混合物Ⅰ和芳香混合物Ⅱ,芳香混合物Ⅰ包括:肉桂醇、肉桂醛、戊基肉桂醛、香叶醇、羟基香茅醛、丁香酚、异丁香酚等;芳香混合物Ⅱ包括:柠檬醛、金合欢醇、己基肉桂醛、香豆素、香茅醇^[8]^。此外,致敏性香料还包括苯甲醇、芳樟醇、水杨酸苄酯、苯甲酸苄酯等^[9]^。除致敏性外,香料使用不当还可导致中毒等其他不良影响^[10,11]^。

目前,化妆品中香料成分的分析研究主要集中于GB/T 24800.10-2009《化妆品中19种香料的测定 气相色谱-质谱法》^[12]^中提到的19种香料,测定的方法也多以气相色谱-质谱法(GC-MS)为主,对茴香醇、甲基丁香酚、异丁香酚甲醚、乙酸丁香酚酯、乙酰基异丁香酚、肉桂酸戊酯、己基肉桂醛、柠檬醛、香豆素的分析测定方法有液相色谱-质谱法(LC-MS)或气相色谱-三重四极杆质谱法(GC-MS/MS)^[13⇓⇓⇓⇓⇓-19]^。GC具有技术和设备成熟、分离效率高、分析速度快、灵敏度高、选择性好及成本低等特点,特别适合易挥发物质的检测,是食品安全分析检测领域普适性好、强有力的检测手段^[20]^。石玲玲等^[21]^利用GC同时检测了化妆品中的苔黑醛、氯化苔黑醛和新铃兰醛。但GC不能直接分析难挥发、热不稳定、强极性、较大相对分子质量的物质^[22]^。质谱法可以弥补色谱法无法分离复杂组分和假阳性的缺点^[23]^,且对未知化合物具有独特的鉴定能力。与单极质谱相比,串联质谱对样品中组成成分的测定更加灵敏^[24]^。谢建军等^[25]^利用分子印迹固相萃取(SPE)-GC-MS对按摩油类化妆品中的16种多环芳烃进行检测,样品经正己烷分散,多环芳烃分子印迹固相萃取柱富集,乙二胺-*N*-丙基硅烷(PSA)固相萃取柱净化,回收率在75.5%~117.2%之间,检出限为0.3~4.1 μg/kg,该方法灵敏度高、结果良好。吴一鑫等^[26]^利用GC-MS/MS测定油类婴儿护理产品中的多环芳烃,样品经环己烷溶解,二甲亚砜萃取,环己烷反萃取,平均回收率在73.3%~125.0%之间,检出限为0.18~3.13 μg/kg,结果较为理想。谢建军等^[27]^利用GC-MS对霜膏类化妆品中16种多环芳烃进行测定,样品经正己烷提取,复合固相萃取柱净化,平均回收率在65.4%~129.7%之间,检出限为0.4~7.9 μg/kg,结果良好。

目前,对于化妆品中同时检测28种香料成分的研究报道较少。Wang等^[28]^通过GC-MS/MS同时测定了化妆品中18种代表性的多环芳烃化合物。Villa等^[29]^采用HPLC同时测定了化妆品中24种香料成分。本方法通过选择化妆品中28种香料成分作为研究对象,以面霜、沐浴露、爽身粉、口红、眼影、润肤露等不同基质的化妆品为试样,选用甲醇进行提取,采用GC-MS/MS外标法进行分析,建立了一种同时分析28种香料成分的检测方法,解决了以往检测难、慢的问题,可节省检测成本和检测时间,提高检测效率,该法准确灵敏,能为化妆品的质量安全提供技术保障。

## 1 实验部分

### 1.1 主要仪器与设备

TQ-8040气相色谱-三重四极杆质谱仪(GC-MS/MS)(配有电子轰击(EI)源,日本岛津公司); ST16R冷冻台式离心机(美国ThermoFisher公司); Supelco 24位固相萃取仪(美国Merck公司); N-EVAP-24氮吹仪(美国Organomation公司); SIGMA3-18KS高速离心机(德国Sigma公司); MTV-100多管漩涡混合仪(杭州奥盛仪器有限公司); MS3圆周振荡器(德国IKA公司); KQ-700DE型数控超声波清洗器(昆山市超声波有限公司); CPA225D电子天平(德国Sartorius公司)。

### 1.2 主要材料与试剂

甲醇、乙酸乙酯(色谱纯,美国Sigma-Aldrich公司);无水硫酸镁、醋酸钠(分析纯,国药集团化学试剂有限公司),PSA(迪马科技有限公司),中性氧化铝小柱(50 mg/3 mL)(Supelco公司)。28种香料成分标准品购自天津阿尔塔公司。

混合标准储备液:分别准确称取28种香料成分标准品各1000 mg置于100 mL容量瓶中,用甲醇溶解并定容至刻度,摇匀,配制成28种香料质量浓度均为10 g/L的混合标准储备液,于4 ℃避光保存,可使用3个月。

### 1.3 样品前处理

#### 1.3.1 固相萃取

称取2 g(精确至0.01 g)试样于10 mL具塞比色管中,加入甲醇准确定容至10 mL,超声提取15 min,上清液待净化。

用5 mL甲醇润洗中性氧化铝柱,取2 mL上述上清液过柱,用10 mL乙酸乙酯洗脱,收集洗脱液缓慢氮吹近干,用2 mL乙酸乙酯定容,振荡混匀装瓶供仪器使用。

#### 1.3.2 QuEChERS

称取2 g(精确至0.01g)试样于10 mL离心管中,加入甲醇准确定容至10 mL,超声提取15 min。加入3 g无水硫酸镁、1 g醋酸钠摇匀,以不低于1200 r/min的转速离心5 min,取上清液2 mL于10 mL离心管中,加入150 mg硫酸镁和50 mg PSA充分振荡,以不低于1200 r/min的转速离心5 min,取上清液上机测定。

### 1.4 仪器条件

#### 1.4.1 色谱条件

色谱柱:Rxi-5sil-Ms 5%苯基二甲基聚硅氧烷石英毛细管柱(30 m×0.25 mm×0.25 μm);升温程序:初始温度80 ℃,保持5 min,以8 ℃/min升温至250 ℃,保持1 min;载气:氦气(He),流速1.2 mL/min;进样量:1 μL;进样方式:不分流进样。

#### 1.4.2 质谱条件

电子轰击离子源;电离能量70 eV;传输线温度:280 ℃;离子源温度:230 ℃;质谱仪接口温度:300 ℃;离子监测模式:多反应监测模式(MRM); 28种香料成分的保留时间、定性离子、定量离子和碰撞能等参数见表1。

**表1:** 28种香料成分的质谱检测参数

No.	Compound	CAS No.	Retention time/min	Product ions (m/z)	Collision energy/eV
1	limonene (柠檬烯)	5989-27-5	5.28	93.00/91.10	12
				93.00/51.10	9
				93.00/77.00^*^	27
2	benzyl alcohol (苯甲醇)	100-51-6	5.28	79.00/51.00	15
				108.00/93.30	21
				79.00/77.10^*^	6
3	linalool (芳樟醇)	78-70-6	7.20	93.00/77.10	6
				69.00/41.10	9
				71.00/43.00^*^	9
4	methyl α-octynoate (α-辛炔酸甲酯)	111-12-6	9.48	79.00/77.00	3
				123.00/77.00	18
				123.00/67.00^*^	9
5	citronellol (香茅醇)	106-22-9	9.98	123.00/81.10	6
				81.00/41.10	3
				69.00/41.00^*^	15
6	geraniol (香叶醇)	106-24-1	10.46	93.00/77.00	12
				69.00/67.10	12
				69.00/41.00^*^	3
7	citral (柠檬醛)	5392-40-5	10.76	69.00/39.00	6
				137.00/109.00	24
				69.00/41.10^*^	6
8	cinnamaldehyde (肉桂醛)	104-55-2	10.88	131.00/103.00	12
				77.00/51.00	9
				103.00/77.10^*^	9
9	hydroxycitronellal (羟基香茅醛)	107-75-5	11.04	81.00/79.10	6
				96.00/79.10	9
				96.00/81.10^*^	15
10	anise alcohol (茴香醇)	105-13-5	11.08	138.00/121.00	18
				138.00/77.00	21
				109.00/77.00^*^	36
11	cinnamyl alcohol (肉桂醇)	104-54-1	11.44	92.00/65.10	6
				134.00/90.80	24
				134.00/92.00^*^	24
12	eugenol (丁香酚)	97-53-0	12.16	164.00/149.00	18
				164.00/90.40	12
				164.00/54.50^*^	18
13	methyl eugenol (甲基丁香酚)	93-15-2	12.84	147.00/91.20	6
				178.00/107.20	9
				178.00/147.10^*^	15
14	coumarin (香豆素)	91-64-5	13.44	118.00/89.90	15
				146.00/88.80	12
				146.00/118.20^*^	33
15	isoeugenol (异丁香酚)	97-54-1	13.56	164.00/77.20	9
				164.00/132.10	36
				77.00/50.90^*^	12
16	α-ionone (紫罗兰酮)	127-41-3	13.92	135.00/78.90	15
				135.00/91.10	9
				107.00/91.00^*^	18
17	methyl isoeugenol (异丁香酚甲醚)	93-16-3	14.16	178.00/107.10	9
				107.00/79.00	9
				178.00/163.10^*^	9
18	eugenol acetate (乙酸丁香酚酯)	93-28-7	14.40	164.00/55.10	6
				164.00/132.10	18
				164.00/149.10^*^	6
No.	Compound	CAS No.	Retention time/min	Product ions (m/z)	Collision energy/eV
19	butylphenyl methylpropional (铃兰醛)	80-54-6	14.61	189.00/91.00	9
				147.00/117.20	18
				189.00/131.00^*^	21
20	acetyl isoeugenol (乙酰基异丁香酚)	93-29-8	15.56	164.00/77.00	24
				164.00/149.20	24
				164.00/103.00^*^	15
21	amyl cinnamate (肉桂酸戊酯)	3487-99-8	16.08	91.00/41.00	27
				91.00/65.00	9
				115.00/63.00^*^	9
22	lyral (新铃兰醛)	31906-04-4	16.22	105.00/77.00	6
				79.00/77.00	30
				107.00/79.20^*^	12
23	α-amylcinnamyl alcohol (戊基肉桂醇)	101-85-9	16.51	91.00/65.00	3
				133.00/115.00	3
				133.00/55.00^*^	15
24	farnesol (金合欢醇)	4602-84-0	16.84	81.00/41.00	6
				67.00/41.00	12
				69.00/41.10^*^	12
25	hexyl cinnamal (α-己基肉桂醛)	101-86-0	17.22	129.00/87.00	21
				115.00/65.00	15
				115.00/89.00^*^	21
26	benzyl benzoate (苯甲酸苄酯)	120-51-4	17.49	77.00/51.10	15
				91.00/65.10	12
				105.00/77.10^*^	9
27	benzyl salicylate (水杨酸苄酯)	118-58-1	18.61	91.00/39.10	15
				91.00/41.10	24
				91.00/65.10^*^	21
28	benzyl cinnamate (肉桂酸苄酯)	103-41-3	20.83	91.00/65.00	12
				131.00/77.10	12
				131.00/103.10^*^	27

* Quantitative ion.

## 2 结果与讨论

### 2.1 样品前处理条件的优化

#### 2.1.1 提取溶剂的选择

针对化妆品样品所选择的提取溶剂主要有乙酸乙酯、乙醇、正己烷、甲醇等。本研究分别考察了甲醇、乙酸乙酯、正己烷作为提取溶剂时对化妆品样品中的香料成分的提取效果。选取沐浴露为基体添加香精香料中烯烃、醇、酚、酯代表性化合物,分别加入甲醇、乙酸乙酯、正己烷至10 mL溶解混匀后,取上清液上机检测,比较三者之间的回收率,结果如图1所示,表明以甲醇作为提取溶剂时,提取效果最好。在文献中,甲醇是化妆品检测中较为常用的提取溶剂^[12,30]^。因此,本研究选用甲醇作为化妆品样品的提取溶剂。

**图1:**
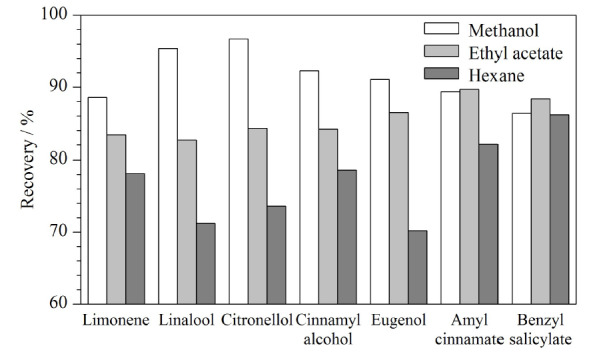
不同提取溶剂对香料的提取效果

#### 2.1.2 提取方式的选择

针对化妆品样品所采用的提取方法有溶剂溶解提取^[30]^、振荡提取^[31]^、蒸馏提取^[32]^、超声提取^[33]^等方式。本研究分别考察了振荡提取、超声提取对化妆品样品中香料成分的提取效果。选择烯烃、醇、酚、酯代表性化合物为分析目标物。结果表明,超声提取效果较振荡提取效果好,且超声提取方式利用超声波的空化作用、机械效应以及热效应等加速待提取物质内有效成分的释放、扩散和溶解,能够显著提高提取效率^[1]^。因此,本研究采用超声提取的方式进行前处理。考察了3种提取时间(5、15、30 min)对化妆品样品中香料成分提取回收率的影响。结果如图2所示,当超声提取15 min和30 min时,其效果相近,且比5 min好。因此,本研究采用提取时间15 min。

**图2:**
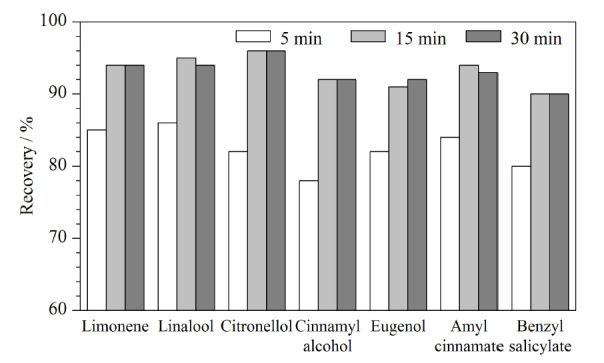
不同超声提取时间下代表性化合物的回收率

#### 2.1.3 固相萃取前处理的优化

李菊等^[34]^报道了GC-MS快速检测乳类化妆品中6种合成麝香,根据其文中阐述,中性氧化铝小柱对化妆品类样品的效果更好,因此本研究采用中性氧化铝小柱作为净化柱,分别使用10 mL乙腈、乙酸乙酯、正己烷进行洗脱,结果如图3所示,发现乙腈极性较强,对弱极性的酯类洗脱能力较差,正己烷属于非极性有机试剂,对于极性较强的醇、酚类洗脱能力较差,乙酸乙酯极性适中,对于28种香料的回收率整体优于乙腈和正己烷,因此洗脱试剂选择乙酸乙酯。考察了固相萃取洗脱液乙酸乙酯的体积(5、10、20 mL),结果表明洗脱体积为20 mL时回收率没有明显提升,洗脱体积为5 mL时回收率较低,因此洗脱时选择使用10 mL乙酸乙酯。

**图3:**
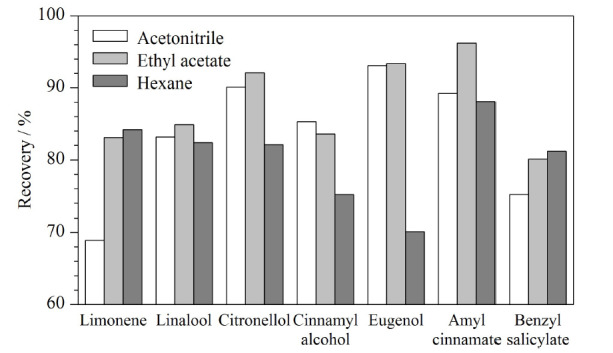
采用不同洗脱试剂时代表性化合物的回收率

#### 2.1.4 QuEChERS前处理的优化

陈岑等^[35]^报道了QuEChERS-HPLC-MS/MS测定祛痘化妆品中7种硝基咪唑类抗菌药物,基于文献中阐述,PSA对目标化合物的回收率影响较小且净化效果好,因此本研究采用PSA并对PSA用量进行了优化,使用色素含量较大的口红作为基体,分别采用20、50、100 mg PSA对提取液进行净化,结果如图4所示,使用50 mg PSA能在确保回收率的情况下达到最好的净化效果。在QuEChERS的前处理过程中,对于不同分取比例进行净化效果研究,采用10 mL甲醇提取样品,分别吸取1、1.5、2、3 mL的上清液进行净化比对。结果表明,分取2 mL上清液对于28种香精有最好的净化效果,得到满足要求的平均回收率。因此,净化时选择分取2 mL上清液进行净化。

**图4:**
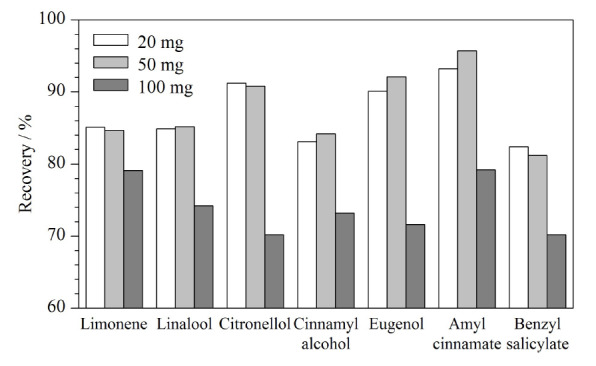
采用不同质量PSA时代表性化合物的回收率

### 2.2 质谱条件的优化

根据GC-MS/MS母离子和子离子一一对应的多反应监测模式,通过设定多个时间段和扫描通道同时分析多种香精香料成分,先通过GC对其进行分离和全扫描,确定香料成分的出峰时间和一级碎片离子,选择离子强度高的一级碎片作为母离子,应用电子轰击扫描模式对母离子在不同碰撞能量下进行二次电离,选择信号较强的二级碎片离子作为子离子,以产生信号强度最强的碰撞能量作为最终优化碰撞能量,优化后的条件见表1,在此条件下,28种香料的总离子流图(TIC)见图5。

**图5:**
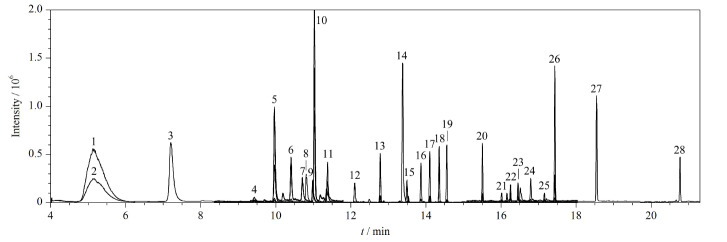
28种香料成分的总离子流图

### 2.3 检出限、定量限与标准曲线

配制系列混合标准溶液,在确定的最佳仪器分析条件下进样测定,以目标组分的峰面积(*Y*)对相应的质量浓度(*X*, μg/L)绘制标准工作曲线,结果表明待测香精香料在线性范围内具有良好的线性关系,线性相关系数(*r*^2^)均大于0.999。分别向空白样品中添加28种香料混合标准溶液,以定量离子信噪比(*S/N*)大于3确定检出限,以*S/N*大于10确定定量限。检出限、定量限及标准曲线见表2,检出限为2~20 μg/kg,定量限为5~50 μg/kg。

**表2:** 28种香料成分的线性方程、相关系数、线性范围、检出限和定量限

No.	Compound	Linear equation	r^2^	Linear range/(μg/L)	LOD/(μg/kg)	LOQ/(μg/kg)
1	limonene	Y=9.02×10^7^X+2.81×10^6^	0.9991	1-100	2	5
2	benzyl alcohol	Y=8.78×10^7^X-3.86×10^5^	0.9992	1-100	2	5
3	linalool	Y=8.47×10^5^X-1.39×10^3^	0.9997	10-1000	20	50
4	methyl α-octynoate	Y=8.41×10^5^X-3.55×10^3^	0.9993	10-1000	20	50
5	citronellol	Y=1.40×10^6^X-1.77×10^3^	0.9991	4-200	7	20
6	geraniol	Y=2.51×10^6^X-7.87×10^5^	0.9991	4-200	7	20
7	citral	Y=2.85×10^6^X+1.03×10^3^	0.9990	4-200	7	20
8	cinnamaldehyde	Y=3.34×10^6^X-8.11×10^3^	0.9992	2-200	3	10
9	hydroxycitronellal	Y=1.01×10^6^X+1.64×10^3^	0.9997	10-1000	20	50
10	anise alcohol	Y=5.89×10^6^X-1.61×10^5^	0.9991	1-100	2	5
11	cinnamyl alcohol	Y=6.51×10^6^X-4.98×10^3^	0.9990	1-100	2	5
12	eugenol	Y=4.53×10^5^X-3.84×10^2^	0.9994	10-1000	20	50
13	methyl eugenol	Y=9.38×10^6^X-2.65×10^4^	0.9993	1-100	2	5
14	coumarin	Y=2.34×10^6^X-3.13×10^4^	0.9991	4-200	7	20
15	isoeugenol	Y=9.60×10^5^X-3.11×10^3^	0.9998	10-1000	20	50
16	α-ionone	Y=1.31×10^6^X+1.13×10^2^	0.9999	4-200	7	20
17	methyl isoeugenol	Y=1.44×10^6^X-4.61×10^4^	0.9991	4-200	7	20
18	eugenol acetate	Y=1.01×10^6^X+6.57×10^3^	0.9991	4-200	7	20
19	butylphenyl methylpropional	Y=4.85×10^5^X-5.07×10^3^	0.9991	10-1000	20	50
20	acety isoeugenol	Y=4.61×10^5^X+1.26×10^4^	0.9990	10-1000	20	50
21	amyl cinnamate	Y=2.37×10^6^X+1.07×10^4^	0.9993	2-200	3	10
22	lyral	Y=1.05×10^6^X+2.36×10^3^	0.9997	4-200	7	20
23	α-amylcinnamyl alcohol	Y=8.05×10^6^X+1.24×10^6^	0.9994	1-100	2	5
24	farnesol	Y=6.11×10^5^X+2.23×10^3^	0.9999	10-1000	20	50
25	hexyl cinnamal	Y=5.30×10^6^X+9.80×10^2^	0.9996	1-100	2	5
26	benzyl benzoate	Y=4.73×10^6^X-5.83×10^4^	0.9991	1-100	2	5
27	benzyl salicylate	Y=2.04×10^6^X-7.22×10^2^	0.9996	4-200	7	20
28	benzyl cinnamate	Y=2.08×10^6^X+3.98×10^4^	0.9999	4-200	7	20

*Y*: peak area; *X*: mass concentration, μg/L.

### 2.4 回收率和精密度

分别在口红(固体)、护肤膏(半固体)、沐浴露(液体)中添加10、20、100 μL质量浓度为10 mg/L的混合标准溶液,使样品中香精的含量为50、100、500 μg/kg。采用固相萃取前处理方式进行加标回收试验,每个水平下做6个平行样。实验结果表明,目标分析物在口红中的回收率为71.3%~120.0%,相对标准偏差(RSD)为1.5%~12.4%;在护肤膏中的回收率为80.4%~120.4%, RSD为3.1%~13.7%;在沐浴露中的回收率为75.6%~110.6%, RSD为4.1%~14.6%。待测香精的添加回收率和RSD见表3。

**表3:** 28种香料成分在3种化妆品基质中的加标回收率(SPE法)(n=6).

No.	Compound	Lipstick								Skin cream						Body lotion					
		50 μg/kg			100 μg/kg			500 μg/kg		50 μg/kg		100 μg/kg		500 μg/kg		50 μg/kg		100 μg/kg		500 μg/kg	
		Rec./ %	RSD/ %		Rec./ %	RSD/ %		Rec./ %	RSD/ %	Rec./ %	RSD/ %	Rec./ %	RSD/ %	Rec./ %	RSD/ %	Rec./ %	RSD/ %	Rec./ %	RSD/ %	Rec./ %	RSD/ %
1	limonene	81.2	10.1		85.4	10.6		87.2	8.6	88.4	11.4	90.5	11.2	85.2	10.2	92.4	10.7	89.4	10.2	92.7	8.4
2	benzyl alcohol	83.4	7.9		84.7	7.6		80.3	6.2	85.2	9.4	83.7	9.6	90.9	8.8	95.2	8.8	94.6	8.1	92.4	5.9
3	linalool	80.2	10.4		80.6	9.5		85.6	8.5	91.6	12.0	93.1	10.5	94.2	9.8	85.6	14.6	86.2	13.5	81.1	11.7
4	methyl *α*-octynoate	103.6	10.7		105.2	9.9		112.4	6.3	114.5	11.2	115.6	11.0	110.9	8.8	102.6	13.4	104.3	11.2	108.2	10.6
5	citronellol	94.2	3.5		97.5	4.5		95.3	2.7	90.5	5.2	91.6	5.5	97.7	4.4	89.1	7.3	90.3	7.5	88.4	6.1
6	geraniol	91.2	2.3		92.3	3.4		95.4	2.2	90.1	5.6	93.7	6.6	92.6	4.2	91.6	8.2	90.2	7.8	94.6	5.3
7	citral	85.6	8.4		89.7	9.0		88.4	6.5	83.7	9.9	88.9	9.5	89.5	7.7	86.2	9.8	89.4	8.9	91.1	7.9
8	cinnamaldehyde	96.0	8.7		98.0	9.2		91.7	6.5	90.0	9.7	91.2	8.2	95.1	7.5	91.1	8.5	93.6	7.8	96.9	6.5
9	hydroxycitronellal	98.1	11.4		102.3	10.6		95.2	8.2	103.2	11.7	105.7	12.1	110.2	10.2	105.6	10.9	102.9	10.3	100.4	8.4
10	anise alcohol	87.5	9.1		88.8	8.7		91.4	7.2	83.5	10.4	90.2	9.5	92.7	8.8	86.5	9.9	88.9	9.0	94.1	8.1
11	cinnamyl alcohol	83.4	7.7		86.4	7.5		88.3	5.4	88.5	9.8	85.4	10.2	90.5	9.0	88.9	11.9	91.4	10.7	93.6	9.4
12	eugenol	90.9	12.4		95.4	11.8		92.6	10.0	99.4	13.7	101.7	12.1	98.5	11.1	91.6	12.9	94.7	11.6	95.1	9.8
13	methyleugenol	105.1	8.4		111.0	7.6		116.5	5.6	101.3	10.6	105.6	9.9	106.7	9.5	98.1	11.7	102.4	10.1	104.1	9.4
14	coumarin	113.1	2.8		114.2	3.5		118.4	2.6	117.4	4.9	117.1	4.8	117.0	3.2	105.3	8.6	109.1	7.9	110.6	5.5
15	isoeugenol	110.5	10.7		105.4	11.4		106.2	9.5	101.8	11.9	102.5	12.4	101.3	10.7	102.7	9.5	104.5	9.1	105.4	8.7
16	*α*-ionone	120.0	7.6		118.4	8.5		111.5	6.4	109.2	9.6	114.1	8.9	112.2	7.5	103.4	11.9	100.6	10.7	109.3	8.1
17	methy isoeugenol	110.4	4.8		105.2	3.5		116.6	3.6	102.7	7.9	100.1	7.5	110.5	6.8	96.2	8.9	97.5	8.1	103.5	7.8
18	eugenol acetate	115.3	8.7		117.4	8.5		118.1	6.9	109.7	10.3	111.9	8.5	105.2	7.8	102.4	13.1	105.6	11.9	109.1	10.1
19	butylphenyl methypropional	113.3	9.7		110.2	10.5		106.2	8.2	107.2	11.4	106.9	11.7	106.2	9.5	98.2	10.7	96.8	9.9	96.9	8.9
20	acety isoeugenol	94.6	10.4		96.4	9.9		99.5	8.7	91.2	10.2	90.3	10.9	89.4	8.9	92.6	10.9	93.1	11.6	96.3	9.6
21	amyl cinnamate	90.7	9.2		95.2	9.3		98.7	7.7	99.1	9.7	102.2	9.5	95.4	8.6	91.2	9.5	89.6	9.1	94.6	7.6
22	lyral	111.5	10.4		113.4	11.5		119.8	9.2	112.4	11.5	115.7	11.3	120.4	9.2	101.2	12.6	105.6	13.6	102.9	10.1
23	*α*-amylcinnamyl alcohol	92.1	5.8		94.4	6.2		90.2	5.0	96.7	5.8	95.1	5.2	91.7	5.0	89.6	7.6	88.9	7.1	91.2	6.0
24	farnesol	78.2	6.3		80.1	5.6		82.5	5.3	85.6	7.3	84.2	5.3	85.3	4.0	75.6	8.9	78.2	8.1	80.3	6.5
25	hexyl cinnamal	95.6	7.1		93.4	7.9		99.4	6.8	89.7	9.2	85.4	9.5	90.3	6.5	91.0	11.4	94.6	10.2	95.6	8.8
26	benzyl benzoate	78.6	1.8		80.5	2.5		82.2	2.0	80.4	4.5	85.2	4.2	84.3	3.1	84.3	5.6	82.6	6.3	85.6	4.1
27	benzyl salicylate	72.5	11.1		75.0	10.4		78.4	9.8	81.6	8.9	80.9	9.5	82.1	6.7	80.1	8.6	86.3	7.9	88.4	4.5
28	benzyl innamate	71.3	1.5		74.3	3.0		77.6	1.5	82.3	3.7	84.7	4.0	87.9	3.5	75.6	8.8	79.3	8.0	80.3	7.7

Rec.: recovery.

### 2.5 两种前处理方式的比较

考虑到市场上化妆品种类繁多,一种前处理净化方式难以满足所有类型化妆品的需要,本文采用2种前处理方式以供选择。SPE前处理方式,净化效果较好,有较强的除油脂的能力,更适用于含油脂较多的种类,但是前处理的时间相对较长。QuEChERS前处理方式,可以有效去除色素,前处理时间较短,对于大批量的样品可以节约前处理的时间。分别在口红(固体)、护肤膏(半固体)、沐浴露(液体)中添加10 μL质量浓度为10 mg/L的混合标准溶液,使样品中香精的含量为50 μg/kg,采用两种前处理方式进行加标回收试验,比较两种前处理的回收率,结果如表4所示。两种方法的回收率均在60%~120%之间,满足实验室质量控制规范技术要求。采用SPE净化的回收率均大于70%,而采用QuEChERS净化的有部分数据小于70%。总体而言,SPE净化比QuEChERS净化效果好,今后的研究可以对QuEChERS净化材料进行进一步优化。

**表4:** SPE和QuEChERS两种前处理方式下目标物的回收率(*n*=3)

No.	Compound	Lipstick			Skin cream			Body lotion	
		SPE	QuEChERS		SPE	QuEChERS		SPE	QuEChERS
1	limonene	80.4	82.7		87.6	89.6		91.3	93.2
2	benzyl alcohol	84.4	61.6		83.2	84.2		73.2	61.6
3	linalool	82.6	93.6		90.4	89.2		86.4	87.3
4	methyl α-octynoate	105.7	100.2		112.5	101.2		106.2	103.6
5	citronellol	92.1	90.3		91.6	80.2		90.3	92.4
6	geraniol	83.5	92.1		82.6	63.4		92.6	90.6
7	citral	86.1	84.3		84.9	65.6		77.4	87.6
8	cinnamaldehyde	96.5	94.6		91.2	89.6		90.6	92.1
9	hydroxycitronellal	87.2	96.2		102.3	84.5		101.2	102.3
10	anise alcohol	86.1	83.4		84.3	76.5		87.6	85.4
11	cinnamyl alcohol	72.2	62.3		87.2	84.6		87.4	76.2
12	eugenol	89.1	92.4		74.6	95.6		90.9	92.6
13	methyleugenol	103.5	66.2		103.6	102.6		99.4	80.2
14	coumarin	107.4	95.6		105.2	112.3		102.6	106.2
15	isoeugenol	106.4	102.2		88.9	100.6		83.5	101.2
16	α-ionone	97.2	114.6		112.3	111.2		106.4	106.9
17	methyl isoeugenol	104.3	102.2		101.3	103.6		98.9	87.2
18	eugenol acetate	111.2	84.2		112.3	103.2		101.2	83.2
19	butylphenyl methylpropional	106.3	109.2		101.6	102.6		98.2	79.4
20	acety isoeugenol	91.5	89.6		94.3	92.6		83.4	93.6
21	amyl cinnamate	98.4	98.4		95.6	98.4		92.6	92.8
22	lyral	114.3	106.8		109.6	106.4		106.2	63.6
23	α-amylcinnamyl alcohol	93.2	90.3		75.2	94.6		90.6	92.4
24	farnesol	76.8	79.6		87.2	86.4		76.9	97.9
25	hexyl cinnamal	94.6	93.2		78.4	90.2		80.6	92.6
26	benzyl benzoate	79.9	61.2		82.3	86.2		85.6	82.3
27	benzyl salicylate	73.6	84.6		80.4	80.3		82.6	71.1
28	benzyl cinnamate	72.4	85.6		81.2	93.1		78.6	76.7

### 2.6 实际样品的测定

按所建立的SPE前处理方法对包括洗发水、沐浴露、润肤露、护肤膏、爽肤水、发膜、按摩油、沐浴盐晶、爽身粉、护肤精油、润唇膏、唇彩、眼影、口红、面膜以及喷雾剂等16种化妆品中的香料成分进行了测定。结果如表5所示,在12种产品中检出目标物,沐浴露中检出柠檬烯、芳樟醇、茴香醇、肉桂醇、丁香酚、甲基丁香酚、香豆素、异丁香酚、紫罗兰酮、异丁香酚甲醚、乙酸丁香酚酯、新铃兰醛、金合欢醇、苯甲酸苄酯、水杨酸苄酯、肉桂酸苄酯;爽身粉中有苯甲醇、芳樟醇、香叶醇、茴香醇、肉桂醇、丁香酚、香豆素、紫罗兰酮、异丁香酚甲醚、乙酸丁香酚酯、苯甲酸苄酯、水杨酸苄酯检出;发膜、眼影、润肤露、唇彩、按摩油、护肤膏、洗发水中均检出香豆素,爽肤水、润唇膏检出柠檬烯。与沈昊宇等^[36]^和刘思然等^[10]^的检出结果相类似。

**表5:** 部分化妆品中检出的香料成分

Cosmetic type	Compounds
Toning lotion	limonene, benzyl alcohol
Hair mask	benzyl alcohol, coumarin, benzyl benzoate
Talcum powder	benzyl alcohol, linalool, geraniol, anise alcohol, cinnamyl alcohol, eugenol, coumarin, α-ionone, methyl isoeugenol, eugenol acetate, benzyl benzoate, benzyl salicylate
Eye shadow	coumarin, α-ionone
Moisturizing lotion	coumarin, α-ionone, butylphenyl methylpropional, benzyl salicylate
Massage oil	coumarin, benzyl benzoate
Lip gloss	coumarin, benzyl salicylate
Skin cream	coumarin
Shampoo	coumarin, farnesol, benzyl benzoate
Body lotion	limonene, linalool, anise alcohol, cinnamyl alcohol, eugenol, methyl eugenol, coumarin, isoeugenol, α-ionone, methyl isoeugenol, eugenol acetate, lyral, farnesol, benzyl benzoate, benzyl salicylate, benzyl cinnamate
Bath salt crystals	linalool, citronellol, citral, α-ionone, farnesol, benzyl benzoate, benzyl salicylate
Lip balm	limonene, benzyl alcohol, linalool

## 3 结论

本研究建立了化妆品中28种香料成分的GC-MS/MS分析方法,针对大部分化妆品提供了2种不同的前处理方式,含油脂比较多的化妆品,比如洗发水、按摩油、护肤精油等采用固相萃取的方式,可以很好地除去油脂;对于色素多、基质复杂如口红、唇彩、眼影等可以采用QuEChERS的方式。该方法准确、简单、可靠、重复性好,适用于各类化妆品中香精香料成分的检测,可为化妆品的质量安全提供技术保障。
